# The Red Blood Cell as a Gender-Associated Biomarker in Metabolic Syndrome: A Pilot Study

**DOI:** 10.1155/2011/204157

**Published:** 2011-09-19

**Authors:** Elisabetta Straface, Lucrezia Gambardella, Antonella Mattatelli, Emanuele Canali, Francesca Boccalini, Luciano Agati, Walter Malorni

**Affiliations:** ^1^Section of Cell Degeneration and Gender Medicine, Department of Therapeutic Research and Medicines Evaluation, Istituto Superiore di Sanità, Viale Regina Elena 299, 00161, Rome, Italy; ^2^Department of Cardiology, “Sapienza” University, 00161, Rome, Italy; ^3^San Raffaele Institute Sulmona, 67039, L'Aquila, Italy

## Abstract

In the present pilot study (56 patients), some red blood cell parameters in samples from patients with metabolic syndrome and subclinical atherosclerosis, but without any sign of coronary artery disease, have been analyzed. The main goal of this work was to determine, in this preclinical state, new peripheral gender-associated bioindicators of possible diagnostic or prognostic value. In particular, three different “indicators” of red blood cell injury and aging have been evaluated: glycophorin A, CD47, and phosphatidylserine externalization. Interestingly, all these determinants appeared significantly modified and displayed gender differences. These findings could provide novel and useful hints in the research for gender-based real-time bioindicators in the progression of metabolic syndrome towards coronary artery disease. Further, more extensive studies are, however, necessary in order to validate these findings.

## 1. Introduction

Metabolic syndrome (MetS) is a cluster of risk factors for atherosclerosis, including insulin resistance, hypertension, glucose intolerance, hypertriglyceridemia, and low high-density lipoprotein-cholesterol (HDL-C) levels [1]. Affected patients have a significantly increased risk of developing atherosclerotic disease, diabetes, and cardiovascular disease (CVD). This is probably due to a blood hypercoagulability as well as to endothelial cell activation. It has been hypothesized that the hypercoagulability state could predispose patients to venous thromboembolism [2]. 

Several epidemiological studies, the Framingham, in particular [3], have investigated into the evolution of cardiovascular disease hypothesizing the presence of a gender difference in the pathogenetic and progression determinants detectable in men and women. For instance, women were found to outlive men and to experience fewer atherosclerotic cardiovascular events, with an incidence lagging behind that in men by 10 to 20 years [4]. This gap in incidence closes with advancing age, when CVD becomes the leading cause of death in women as well as in men [5, 6]. In consideration of the high incidence of morbidity and mortality, due to CVD, and of the paucity of well-established gender-associated markers, further studies focused at identifying novel bioindicators should be considered as mandatory.

On these bases, a pilot study has been conducted in a low number of patients with MetS of both sexes and subclinical atherosclerosis with the aim to identify innovative peripheral blood biomarker in this preclinical phase [7]. We focused our attention on the red blood cell (RBC) as a candidate possibly implicated in these pathologic conditions. RBCs are peculiar cells aimed at the delivery of oxygen and nitric oxide to the periphery and carbon dioxide to the lungs. In addition, they also exert, under physiological conditions, a scavenging activity towards reactive oxygen and nitrogen species often overproduced in morbidity states, for example, in inflamed tissues. Their deformability, essential for their circulation in small blood vessels, is an important prerequisite for such vascular “antioxidant” functions. Conversely, when the redox state of RBCs is altered, erythrocytes can turn out to be a source of reactive species, and, consequently, its typical structural and functional features are lost [8, 9]. Importantly, the oxidatively modified erythrocyte increases its aggregability and adhesiveness to the endothelium and to other blood cells, thus contributing to vascular damage. In addition, CVD risk factors, namely, insulin resistance, obesity, and hypertension, all share a common abnormal ion profile in RBCs. This might help to explain their frequent clinical coexistence. Specifically, it has been hypothesized that RBC intracellular pH levels are lower and inversely linked to both body mass index (BMI) and fasting insulin concentrations either in normotensive or hypertensive individuals. Moreover, ionic imbalance, for example, of intracellular potassium, magnesium, and calcium, can decrease intracellular pH levels also resulting in a reduced GSH/GSSG ratio [10]. In this work, three different putative “indicators” of RBC injury and aging have been evaluated: glycophorin A (GA), CD47, and phosphatidylserine (PS). The first is a glycoprotein that is widely expressed at the surface of RBC and is downregulated during senescence [11]; the second, CD47, as for other cells, is an integrin-associated protein that acts as a “marker of self” [12]; the third is a phospholipid localized to the inner leaflet of the plasma membrane, which is externalized to the outer leaflet during cell remodeling leading to cell death, for example, by eryptosis [13, 14]. Notably, it has been reported that phosphatidylserine-exposing RBCs may adhere to the vascular walls [14] and may interfere with microcirculation as it has been proposed to occur in the metabolic syndrome [9]. Importantly, GA loss, PS externalization (evaluated in terms of positivity to its ligand: annexin V), and reduced expression of CD47, respectively, have been reported as critical events responsible for the removal of RBCs at the end of their lifespan [15–17].

## 2. Patients and Methods

### 2.1. Study Population

The study population consisted of 56 ambulatory subjects with MetS (31 men and 25 women, aging 50–70 years) and 40 age-matched healthy donors (HDs) (22 men and 18 women). All patients and HDs were Caucasian. All study subjects underwent a complete cardiovascular evaluation which has included: history and physical examination, heart rate, blood pressure, fasting serum glucose; fasting plasma lipids, Fibrinogen, CRP, comprehensive two-dimensional echocardiogram, carotid echo-color Doppler and exercise ECG testing. Healthy donors were identified on the basis of the absence of CVD risk factors and a completely normal CVD screening.

MetS was diagnosed according to the amended National Cholesterol Education Program's Adult Treatment Panel III (ATP-III) Guidelines in individuals meeting three or more of the criteria reported elsewhere [1]. Healthy donors were identified on the basis of the absence of CVD risk factors and a completely normal CVD screening. We included in the study (i) patients with an increased (N1 mm) carotid intima-media thickness (IMT), but in the absence of known or suspected coronary artery disease (CAD), and (ii) only women in postmenopausal and without hormone replacement therapy. The patient characteristics have been reported in Table 1.

Patients with previous myocardial infarction, previous coronary artery by-pass graft, coronary angioplasty or positive exercise ECG testing, depression, inflammatory diseases, and ACEI treatment were excluded from the study. The nature and the purpose of the study were explained to all participants who gave their informed consent following the rules of good medical practice. This study was approved by the Institutional Review Board of “Sapienza” University of Rome (Italy). The investigation was conformed to the principles outlined in the Declaration of Helsinki. 

### 2.2. Isolation of Erythrocytes

Human erythrocyte suspensions were prepared from fresh venous blood collected as previously reported [11].

### 2.3. Analysis of the Redox Balance in RBCs

For intracellular ROS production, RBCs (5 × 10^5^ cells) were incubated in Hanks' balanced salt solution (HBSS, pH 7.4) containing dihydrorhodamine 123 (DHR 123, Molecular Probes, USA). Intracellular content of reduced thiols was explored by using 5-chloromethylfluoresceindiacetate (CMFDA, Molecular Probes). Samples were then analyzed with a FACScan flow cytometer (Becton Dickinson, Mountain View, Calif, USA). The median values of fluorescence intensity histograms were used to provide semiquantitative evaluation of reduced thiols content and reactive oxygen species (ROS) production.

### 2.4. Evaluation of RBC Injury

Quantitative evaluation of RBCs with phosphatidylserine externalization [11] was performed by flow cytometry after double staining using FITC-conjugated annexin V and 0.05% Trypan blue for 10 min at room temperature and analyzed by fluorescence-activated cell sorting (FACS) in the FL3 channel to determine the percentage of dead cells.

### 2.5. Quantitative and Qualitative RBC Protein Analyses

For glycophorin A detection, RBCs were stained with anti-glycophorin A (Saint Louis, Mo, USA) monoclonal antibodies and subsequently incubated with anti-mouse IgG-fluorescein-linked whole antibodies (Sigma). For CD47, RBCs were fixed with 4% paraformaldehyde, permeabilized with 0.5% Triton X-100 (Sigma Chemical Co, Mo, USA), stained with monoclonal anti-CD47 (Santa Cruz Biotechnology, CA, USA), and subsequently incubated with antimouse IgG-fluorescein-linked whole antibody (Sigma). Finally, all the samples were analyzed with a FACScan flow cytometer or observed with a Nikon Microphot fluorescence microscope.

### 2.6. Morphometric Analyses

Whole blood from MetS patients and healthy donors was stripped on the slide, dried at room temperature, and observed by light or differential interference contrast (DIC) microscopy. Altered erythrocyte shape was evaluated by counting at least 500 cells (50 RBCs for each field at a magnification of 1500x) from MetS patients and healthy donors.

### 2.7. Statistical Analyses

Cytofluorimetric results were statistically analyzed by using the nonparametric Kolmogorov-Smirnov test using Cell Quest Software. A least 20,000 events were acquired. The median values of fluorescence intensity histograms were used to provide a semiquantitative analysis. Statistical analyses of collected data were performed by using Student's *t*-test. 

## 3. Results

### 3.1. Redox Balance

**Figure 1:**
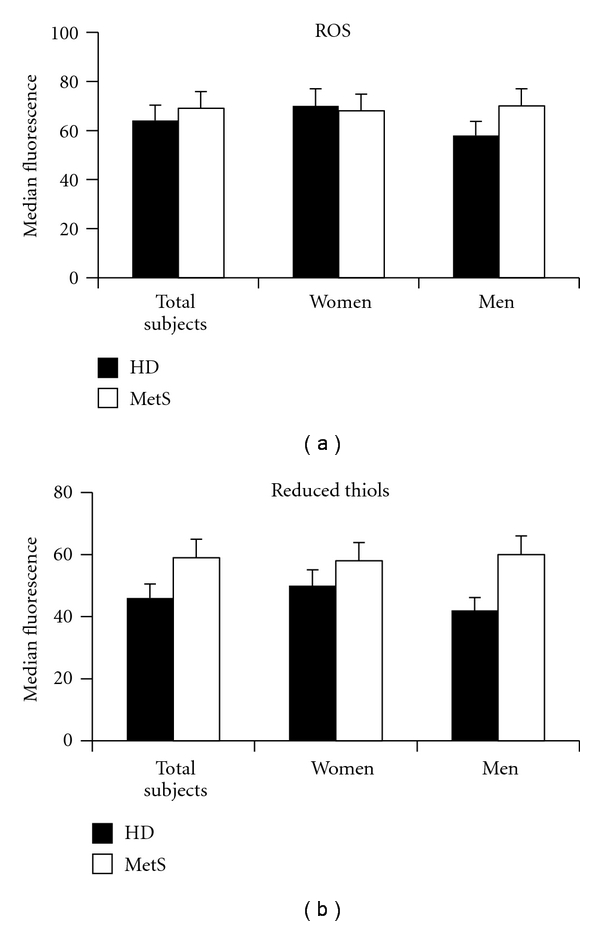
Cytofluorimetric analysis of redox balance in the RBCs. Histograms representing flow cytometry analysis of (a) ROS production and (b) intracellular content of reduced thiols. The numbers are the mean ± SD of 40 HD (22 male and 18 female) and 56 MetS patients (31 male and 25 female). No significant changes were detectable in the ROS production and the GSH presence in RBCs from MetS patients in comparison with that from healthy donors. No gender differences were observed.

Considering that changes in the redox state can contribute to the loss of RBC structure and function [8], two important parameters have been analyzed. We measured the reactive oxygen species (ROS) production and the total thiol content (essentially referred to as reduced glutathione). However, no significant differences were detected in the ROS and total thiol production in RBCs from patients with MetS in comparison with that from healthy donors (Figures 1(a) and 1(b)). Furthermore, no gender differences were observed.

### 3.2. Morphological Analyses

**Figure 2:**
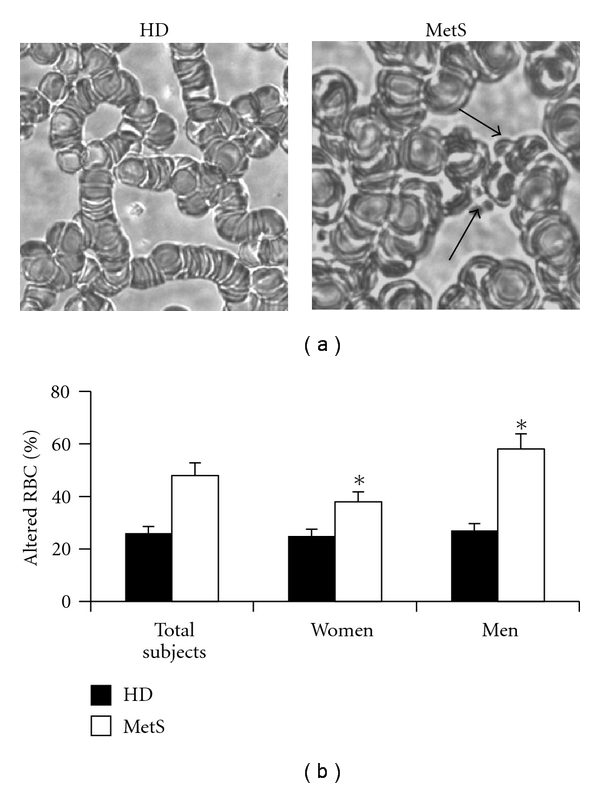
Morphological analyses of RBCs. (a) Two representative micrographs obtained by light microscopy displaying morphological alteration in RBCs from MetS patients (right panel) with respect to those from healthy donors (left panel). Note the different aggregation features and the presence of surface protrusions (arrows). (b) Morphometric analysis indicating the percentage of altered RBCs in both HD and MetS patients. Data are the mean ± SD of 40 HD (22 male and 18 female) and 56 MetS patients (31 male and 25 female). When morphometric analyses were performed as stated in Methods, a gender difference in terms of morphological alteration was detectable in MetS patients. *P < 0.01, men with MetS versus women with MetS.

Changes of RBC viscosity, adhesiveness, and aggregability have been detected in many human pathologic conditions. In particular, changes of erythrocyte adhesiveness/aggregation and morphology have been proposed as useful markers to detect inflammatory conditions, plaque instability, and atheroma progression in patients with coronary artery disease [18–20]. In order to determine whether RBCs could be considered as biomarkers of diagnostic or prognostic value in metabolic syndrome, their morphology and adhesiveness/aggregation properties were studied in cells from both healthy donors and in patients with MetS. These analyses were carried out by means of light microscopy (two representative micrographs are shown in Figure 2(a)) and DIC microscopy evaluations (not shown). A significant increase of RBCs displaying morphological alterations has been detected in samples obtained from patients with MetS with respect to that from healthy donors (Figure 2(b); note that the percentage of altered RBCs, about 20%, was within the normal values) [21]. In particular, RBCs with numerous surface protrusions (acanthocytes) have been detected (arrows). Moreover, when patient gender was taken into consideration, a difference in terms of morphological alterations was detected. In particular, the percentage of altered RBCs was higher in men than in women with MetS. As expected, no gender difference was detected in RBCs from healthy donors (black histograms in Figure 2(b)).

### 3.3. RBC Senescence and Death

**Figure 3:**
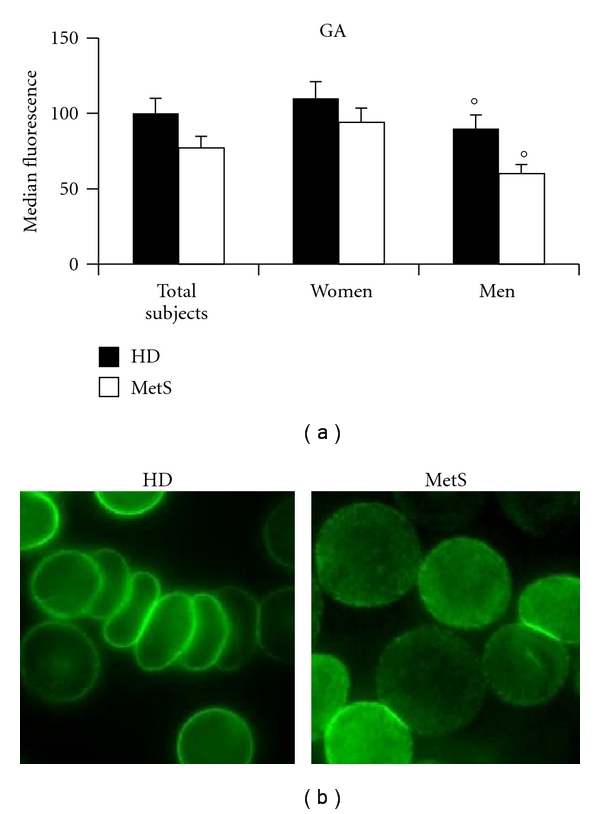
Glycophorin A analysis. (a) Histogram representing flow cytometry evaluations of glycophorin A. The numbers represent the mean ± SD of 40 HD (22 male and 18 female) and 56 MetS patients (31 male and 25 female). No changes, in terms of GA expression, were detectable in RBCs from MetS patients with respect to those from healthy donors. Conversely, a significant (°*P* < 0.05) decrease of this protein was evident in RBCs from men with MetS versus healthy male donors. (b) Representative immunofluorescence micrographs displaying different arrangement and positivity for GA of RBCs from a healthy man and a man with MetS.

**Figure 4:**
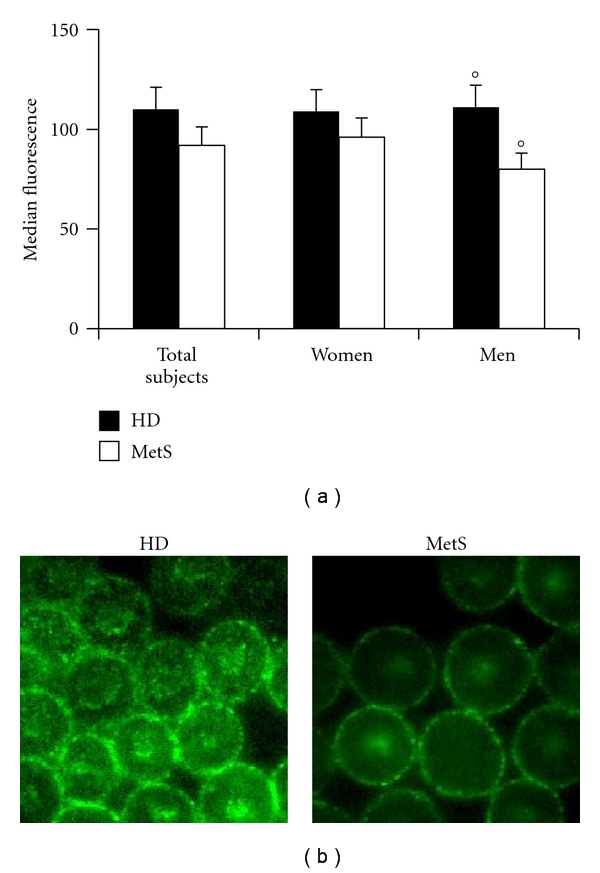
CD47 analysis. (a) Histogram representing flow cytometry evaluations of CD47. The numbers represent the mean ± SD of 40 HD (22 male and 18 female) and 56 MetS patients (31 male and 25 female). No changes, in terms of CD47 expression, were detectable in RBCs from MetS patients with respect to those from healthy donors. Conversely, a significant (°*P *< 0.05) decrease of this protein was evident in RBCs from men with MetS versus healthy male donors. (b) Representative immunofluorescence micrographs displaying different arrangement and positivity for CD47 of RBCs from a healthy man and a man with MetS.

**Figure 5:**
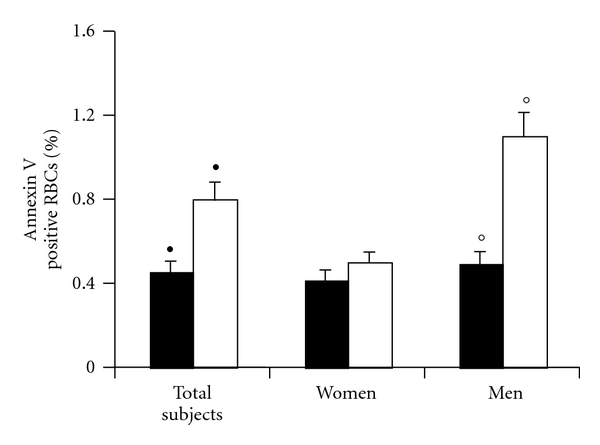
Phosphatidylserine externalization. Histograms representing the percentage of annexin-V-positive RBCs. Data are the mean ± SD of 40 HDs (22 male and 18 female) and 56 MetS patients (31 male and 25 female). An increased positivity was detectable in RBCs from MetS patients with respect to those from healthy donors (*λ*) ^•^ *P* < 0.05. Analyzing separately RBCs from males and females, a significant increased positivity was detectable only in RBCs from males with MetS with respect to healthy males. °*P* < 0.01.

The analysis of GA and CD47 was carried out by both flow cytometry (Figures 3(a) and 4(a)) and immunofluorescence microscopy (Figures 3(b) and 4(b)). These analyses clearly demonstrated that the expression of GA and CD47 was substantially similar in RBCs from patients with MetS and in those from healthy donors (Figures 3(a) and 4(a)). However, when cells from males and females were analyzed separately, the expression of these molecules was found significantly (*P* < 0.05) different: lower in RBCs from men with MetS in comparison with RBCs from women with MetS. Representative immuno-fluorescence micrographs displaying GA and CD47 positivity in RBCs from healthy donors and men with MetS are shown in Figures 3(b) and 4(b). Moreover, as concerns PS externalization, a significantly higher translocation of PS to the outer plasma membrane leaflet was detected in RBCs from patients with MetS in comparison with those from HD (Figure 5). Furthermore, a gender difference was also appreciable. In fact, in RBCs from male patients with MetS, the surface positivity for PS was twice that of controls, whereas no difference, in term of PS externalization, has been detected between RBCs from HD females and RBCs from females with Mets.

## 4. Discussion

In the present pilot study, we focused our attention on the red blood cell as candidate biomarker of metabolic syndrome. We also investigated about possible gender differences. We analyzed in detail three different “indicators” of RBC injury and aging: glycophorin A, CD47, and phosphatidylserine. To this aim, diverse blood determinants were evaluated in a low number (*n* = 56) of male and female patients that did not display any sign of CAD. To be included in the study, these patients should have at least 3 major criteria for MetS and a pathologically abnormal carotid IMT. Carotid IMT was in fact linked to many cardiovascular outcomes, including cerebral and coronary events, and it has been proposed as an index of subclinical atherosclerosis [22]. From a clinical point of view, despite a similar incidence of risk factors and IMT, men with MetS showed a significantly higher LV function and structure involvement in the absence of patent CAD symptoms. 

It is a matter of fact that circulating erythrocytes could contribute to the pathogenesis of cardiovascular diseases [2, 8, 23]. The shape maintenance as well as mechanical deformability and elasticity of RBCs (7 *μ*m) is essential pre-requisites for their circulation, specifically in small blood vessels (about 5 *μ*m). If the RBC is altered, its aggregability and adhesive properties change, thus contributing to vascular damage [23]. In our study, we detected differences, in terms of cell aging, cell adhesion, and/or aggregation, in RBCs from MetS patients with respect to those of healthy donors. In particular, we observed that the ability to pile was modified in erythrocytes either from women or from men with MetS. Moreover, they appeared to increase their adhesiveness to a substrate. This was probably associated with the increased PS externalization, a well-known marker of RBC aging and death, that has been associated with increased cell adhesion properties. In fact, studies have shown that RBC injury due to energy or antioxidant depletion causes breakdown of membrane phosphatidylserine asymmetry, with consequent exposure of phosphatidylserine at the erythrocyte surface (eryptosis) and binding to the phosphatidylserine receptors at macrophages and liver Kupffer cells, which then engulf and degrade the affected RBCs [14]. Locally, these phagocytic cells produce superoxide anion, which activates NF-*κ*B and c-JN, inflammatory signalling pathways that regulate cellular transcriptional events, thereby leading to greater production of TNF-*α*, IL-6, and other proinflammatory mediators [9]. This could be of relevance in the light of recent works that describe the disparity of vascular cells from males and from females in terms of their “basal” redox state and their susceptibility to oxidative stress [24, 25] contributing to the pathogenesis of vascular diseases [26]. 

Interestingly, as concerns gender, we also found significant differences (morphological alterations, aging-associated molecules GA, and adhesion-associated molecules CD47 and PS). These results are in accord with several literature data [23] that suggest RBC as real-time biomarkers of disease progression and pathogenetic determinants in cardiovascular diseases. RBCs can in fact contribute to atherosclerotic plaque formation [19] and can behave as prooxidants, thus contributing to the pathogenetic mechanisms of vascular diseases [8]. For example, it has been demonstrated that oxidized erythrocytes can represent potential sources of systemic inflammation: the increase of exogenous or endogenous CO_2_ deoxygenates haemoglobin favouring the formation of methemoglobin [9]. 

Altogether the results of this pilot study are in line with the literature data indicating erythrocytes as possible biomarkers of vascular diseases [23]. We also hypothesize that gender could represent a key variable in this issue [3]. Further studies appear, however, as mandatory in order to assess if the gender-specific biomarkers analyzed here could be detected in a larger study population, thus providing useful insights for a gender-based management of MetS progression.

## Figures and Tables

**Table 1:** Patients' characteristics. Significant differences are in bold. Data are the mean ± SD of 56 MetS patients (31 male and 25 female) and 40 HD (22 male and 18 female).

Variables	56 MetS (*M *= 31 *F* = 25)	40 HD (*M* = 22 *F* = 18)	*P*-values
*Risk factors*			
Body mass index (Kg/m^2^)	**31.98 ± 4.84**	**21.05 ± 2.01**	**0.0001**
Waist circumference (cm)	**112.7 ± 14.98**	**71.79 ± 6.22**	**0.0001**
Systolic blood pressure (mmHg)	**134.57 ± 17.7**	**120.63 ± 8**	**0.03**
Diastolic blood pressure (mmHg)	**84.13 ± 9.37**	**74.95 ± 5.68**	**0.001**
Glucose (mg/dl)	**122.71 ± 34.87**	**63.84 ± 9.38**	**0.001**
Total cholesterol (mg/dl)	197.62 ± 38.83	178.39 ± 20.23	0.15
LDL-cholesterol (mg/dl)	125.4 ± 36.08	114.27 ± 28.51	0.31
HDL-cholesterol (mg/dl)	44.41 ± 8.47	47.09 ± 8.8	0.78
Triglyceride (mg/dl)	144.8 ± 72.28	118.94 ± 48.96	0.26
Family history of CAD	12 (50%)	4 (21%)	0.60
Family history of diabetes	13 (54%)	5 (26%)	0.80
Currently smokers	5 (21%)	12 (63%)	0.67
*Echocardiography parameters*			
LVEF (%)	**52.46 ± 6.16**	**59.68 ± 2.82**	**0.001**
SIV (mm)	**11.58 ± 0.92**	**8.74 ± 1.28**	**0.0001**
PP (mm)	**11.13 ± 0.90**	**9.21 ± 1.22**	**0.001**
LVEDV (mL)	**126.78 ± 31.19**	**115.00 ± 18.93**	**0.05**
LVESV (mL)	**58.57 ± 17.20**	**40.05 ± 6.32**	**0.002**
LVM-I (g)	**114.25 ± 16.84**	**77.36 ± 31.24**	**0.0001**
*Carotid echo-color Doppler parameters*			
CCA Sx (mm)	**1.25 ± 0.36**	**0.73 ± 0.22**	**0.0001**
ICA Sx (mm)	**1.68 ± 0.73**	**0.67 ± 0.26**	**0.001**
CCA Dx (mm)	**1.30 ± 0.39**	**0.56 ± 0.28**	**0.001**
ICA Dx (mm)	**1.96 ± 0.79**	**0.66 ± 0.27**	**0.0001**

## References

[1] Grundy SM, Cleeman JI, Daniels SR (2005). Diagnosis and management of the metabolic syndrome. An American Heart Association/National Heart, Lung, and Blood Institute scientific statement: expert panel on detection, evaluation, and treatment of high blood cholesterol in adults. Executive summary. *Circulation*.

[2] Ay C, Tengler T, Vormittag R (2007). Venous thromboembolism—a manifestation of the metabolic syndrome. *Haematologica*.

[3] Ingelsson E, Pencina MJ, Tofler GH (2007). Multimarker approach to evaluate the incidence of the metabolic syndrome and longitudinal changes in metabolic risk factors. The Framingham Offspring Study. *Circulation*.

[4] Mølstad P (2009). Coronary heart disease in women: less extensive disease and improved long-term survival compared to men. *Scandinavian Cardiovascular Journal*.

[5] Kannel WB (2002). The Framingham Study: historical insight on the impact of cardiovascular risk factors in men versus women. *Journal of Gender-Specific Medicine*.

[6] Legato MJ, Gelzer A, Goland R (2006). Gender-specific care of the patient with diabetes: review and recommendations. *Gender Medicine*.

[7] Prandoni P (2007). Links between arterial and venous disease. *Journal of Internal Medicine*.

[8] Minetti M, Agati L, Malorni W (2007). The microenvironment can shift erythrocytes from a friendly to a harmful behavior: pathogenetic implications for vascular diseases. *Cardiovascular Research*.

[9] Zappulla D (2008). Environmental stress, erythrocyte dysfunctions, inflammation, and the metabolic syndrome: adaptations to CO2 increases?. *Journal of the Cardiometabolic Syndrome*.

[10] Resnick LM (1993). Ionic basis of hypertension, insulin resistance, vascular disease, and related disorders. The mechanism of “syndrome X”. *American Journal of Hypertension*.

[11] Matarrese P, Straface E, Pietraforte D (2005). Peroxynitrite induces senescence and apoptosis of red blood cells through the activation of aspartyl and cysteinyl proteases. *FASEB Journal*.

[12] Khandelwal S, Van Rooijen N, Saxena RK (2007). Reduced expression of CD47 during murine red blood cell (RBC) senescence and its role in RBC clearance from the circulation. *Transfusion*.

[13] Mandal D, Mazumder A, Das P, Kundu M, Basu J (2005). Fas-, caspase 8-, and caspase 3-dependent signaling regulates the activity of the aminophospholipid translocase and phosphatidylserine externalization in human erythrocytes. *Journal of Biological Chemistry*.

[14] Lang F, Gulbins E, Lang PA, Zappulla D, Föller M (2010). Ceramide in suicidal death of erythrocytes. *Cellular Physiology and Biochemistry*.

[15] Daugas E, Candé C, Kroemer G (2001). Erythrocytes: death of a mummy. *Cell Death and Differentiation*.

[16] Pietraforte D, Matarrese P, Straface E (2007). Two different pathways are involved in peroxynitrite-induced senescence and apoptosis of human erythrocytes. *Free Radical Biology and Medicine*.

[17] Oldenborg PA, Zheleznyak A, Fang YF, Lagenaur CF, Gresham HD, Lindberg FP (2000). Role of CD47 as a marker of self on red blood cells. *Science*.

[18] Matsuda R, Kaneko N, Kikuchi M (2003). Clinical significance of measurement of plasma annexin V concentration of patients in the emergency room. *Resuscitation*.

[19] Modun D, Music I, Vukovic J (2008). The increase in human plasma antioxidant capacity after red wine consumption is due to both plasma urate and wine polyphenols. *Atherosclerosis*.

[20] Kyrle PA, Hron G, Eichinger S, Wagner O (2007). Circulating P-selectin and the risk of recurrent venous thromboembolism. *Thrombosis and Haemostasis*.

[21] Lucantoni G, Pietraforte D, Matarrese P (2006). The red blood cell as a biosensor for monitoring oxidative imbalance in chronic obstructive pulmonary disease: an ex vivo and in vitro study. *Antioxidants and Redox Signaling*.

[22] Lorenz MW, Markus HS, Bots ML, Rosvall M, Sitzer M (2007). Prediction of clinical cardiovascular events with carotid intima-media thickness: a systematic review and meta-analysis. *Circulation*.

[23] Berliner S, Rogowski O, Aharonov S (2005). Erythrocyte adhesiveness/aggregation: a novel biomarker for the detection of low-grade internal inflammation in individuals with atherothrombotic risk factors and proven vascular disease. *American Heart Journal*.

[24] Malorni W, Straface E, Matarrese P (2008). Redox state and gender differences in vascular smooth muscle cells. *FEBS Letters*.

[25] Straface E, Vona R, Gambardella L (2009). Cell sex determines anoikis resistance in vascular smooth muscle cells. *FEBS Letters*.

[26] Matarrese P, Colasanti T, Ascione B Gender disparity in susceptibility to oxidative stress and apoptosis induced by autoantibodies specific to RLIP76 in vascular cells.

